# A comprehensive analysis of carbonate matrix acidizing using viscoelastic diverting acid system in a gas field

**DOI:** 10.1038/s41598-024-52104-5

**Published:** 2024-01-17

**Authors:** Mostafa Keihani Kamal, Javad Mahdavi Kalatehno, Peyman Daneshfar, Fatemeh Yousefmarzi

**Affiliations:** 1 Department of Petroleum Engineering, Amirkabir University of Technology, Tehran, Iran; 2 Dana Energy Company, Tehran, Iran

**Keywords:** Energy science and technology, Engineering

## Abstract

This paper explores matrix acidizing, a method to enhance well productivity by injecting acid into the formation to dissolve damage or create flow channels. Focusing on gas well acidizing, it introduces a groundbreaking three-stage approach with hydrochloric acid (HCl) and viscoelastic diverting acid (VDA). Unlike recent research, which often overlooked specific VDA stages and favored VES or surfactant gelled systems, this study innovatively integrates VDA throughout laboratory experimentation, simulation modeling, and operational execution. The article showcases the effectiveness of HCl and VDA in dissolving reservoir materials, preventing issues like emulsion formation and iron precipitation, reducing corrosion and H2S emissions, enhancing penetration depth, fluid flow channels, and stimulating all reservoir layers. Utilizing a numerical model, it recommends an optimal acidizing method with five main acid injection stages and five VDA injection stages. The results demonstrate a notable increase of 100% in gas production, an 84% rise in gas pressure, and a reduction of BS&W from 7 to 3%. Aimed at industry professionals, this paper serves as a guide for optimizing well productivity and gas recovery processes.

## Introduction

In the oil and gas industry, effective reservoir stimulation is crucial for enhancing well productivity^1,2^. Acidizing, a common technique for achieving this, involves injecting an acid system into the formation below fracturing pressure^3,4^. The acid system serves to dissolve formation damage or create new flow channels, ultimately increasing the permeability of the wellbore region. Matrix acidizing, a specific application, targets pressure loss reduction and production rate increase^5^. This method aims to eliminate or prevent damaged zones near the wellbore^6,7^. Hydrochloric acid (HCl) is widely employed in carbonate matrix acidizing due to its reactivity with calcite and dolomite, coupled with its cost-effectiveness^8,9^. Although HCl effectively dissolves minerals, its rapid consumption poses challenges^9^. Additionally, substances like viscoelastic diverting acid (VDA) are used for optimal performance in acidizing operations^10–12^. VDA, a formulation combining a viscoelastic surfactant, acid, solvent, and other additives, exhibits unique rheological properties^13^. The viscoelastic nature of VDA, allowing it to alter viscosity with shear rate, stands out as one of its most significant properties. At rest, VDA possesses high viscosity, enabling effective fluid diversion and control during acid injection. However, when exposed to shear forces, such as during pumping, its viscosity decreases, facilitating the smooth movement of fluids through the wellbore and into the formation. VDA excels at redirecting acid flow to desired reservoir formation zones^14^. In regions characterized by high permeability, the viscoelastic properties of VDA facilitate the formation of transient high-viscosity bridges or barriers. These structures redirect acid flow into previously untreated areas, ensuring a more uniform and comprehensive stimulation treatment. Through selective acid redirection, VDA enhances reservoir clearance efficiency and prevents the over-treatment of already stimulated areas^15–17^. The selection of acid additives in both acid and VDA designs is guided by considerations of formation damage and reservoir fluids^18,19^. However, simultaneous use of multiple additives poses the risk of increased incompatibility or unforeseen interactions with formation or reservoir fluids, potentially resulting in unintended formation or reservoir damage. Consequently, a one-size-fits-all acid and additive formula for all reservoir types is non-existent, given the inherent variations in formation mineralogy and fluid composition between reservoirs^9^. Therefore, a thorough evaluation of various commercial inhibitors, surfactants, and solvents becomes imperative to better understand the effects of these additives on reservoir acidizing. In accordance with their functions, acid additives can be classified into categories such as corrosion inhibitors, iron control agents, emulsion inhibitors, H_2_S scavengers, and more^20,21^.

Matrix acidizing is a crucial technique in the oil and gas industry that aims to enhance well productivity by decreasing formation damage and enhancing fluid flow within the formation matrix^22–24^. Ongoing research and engineering endeavors delve into innovative matrix acidizing operations, fluid compositions, and diversion techniques. In this dynamic landscape, various studies have made substantial contributions, shedding light on diverse aspects of matrix acidizing and paving the way for improved practices. Al-Dhafeeri et al. (2002) conducted a study in Saudi Arabia on real-time evaluation of matrix acidizing efficiency using skin effect analysis. They used Paccaloni's and Prouvost-Economides' methods to monitor skin factors during acid jobs, resulting in lower operational costs and increased stimulation returns^25^. Derradji et al. (2003) optimized matrix acidizing treatment strategies for the Haoud Berkaoui Field in Algeria, addressing mud invasion-induced formation damage. They found the optimal fluid composition, specifically acid system 3, improves permeability while minimizing emulsion or precipitation risks, emphasizing the importance of laboratory experiments^26^. Enelamah et al. (2003) provided a critical perspective on matrix acidizing in Niger Delta sandstone formations, emphasizing the significance of sound engineering practices in navigating uncertainties^27^. Patton et al. (2003) studied matrix acidizing strategies for the Monterey Formation and found that HF acidizing is effective due to its compatibility with siliceous materials and ability to cause damage. They also highlighted the effectiveness of bullheading with foam diversion, highlighting its potential for productivity improvement and highlighting the potential of various acid systems and techniques^28^. Haldar et al. (2004) created the innovative In-Situ Cross-linking Acid Diverting Agent (ISCADA), which momentarily obstructs high-permeability or undesirable layers, enabling customized breaking times for effective treatment^29^. Nasr-El-Din et al. (2004) devised an ingenious method for acidizing horizontal wells, thus decreasing the risk of drilling mud filter cake and increasing production in Saudi Arabian carbonate reservoirs^30^. O'Driscoll et al. (2005) emphasized the importance of effective matrix acidizing in horizontal wells, despite the obstacles of acid quality, equipment accessibility, and treatment design^31^. Kasza et al. (2006) introduced emulsified acid treatments for high-temperature, low-permeability dolomite formations, focusing on their economic viability and potential for mitigating wellbore damage^32^. "Large Liquid Volume Deep Penetration Acidizing" was introduced by Gao et al. (2019) as a new method to increase carbonate reservoir production by creating long-lasting wormholes and enhancing stimulation efficiency^33^. Al-Rekabi et al. (2020) conducted a study on skin analysis to optimize carbonate matrix acidizing efficiency. They focused on fluid composition optimization, minimizing induced damage, and using real-time skin trend monitoring models. Their findings showed an average added skin value of + 3 to + 10 and a stimulation efficiency of 30% to 40%^34^. The 2022 research by Sierra et al. explores the optimization of stimulation processes in Mexico's "T" field using single-phase retarded acid. The study found that this method significantly increased production by 25% in selected wells, surpassing the performance of organic acid treatments. The study also highlighted the system's wormhole regime, solubility advantages, and controlled reaction rates, offering operational flexibility and increased system stability^35^. The 2023 study by Morrow et al. explores the effective stimulation of a horizontal water injection well with Limited Entry Liner (LEL) completion in a Middle East carbonate reservoir. They use a novel approach involving a viscoelastic diverter acid (VEDA) fluid system, which enhances injectivity and distributes water evenly across the horizontal drain, especially in cases with significant permeability contrasts between well compartments^36^.

This paper presents a groundbreaking exploration of gas well acidizing in southern Iran, focusing on the innovative combination of Hydrochloric Acid (HCl) and Viscoelastic Diverting Acid (VDA) systems. The study follows a distinctive three-stage methodology, encompassing laboratory experiments, simulation modeling, and operational execution.

In the initial phase, the laboratory development of the main acid and VDA systems is detailed, incorporating various additives. The selection criteria for effective additives and their concentrations are meticulously outlined, ensuring the desired acid composition for the well. Subsequently, the simulation stage utilizes software tools to replicate the acid job, injection method, and critical operating parameters. This simulation aims to provide insights into the acidizing procedure, optimizing the injection method based on the field's unique characteristics.

The third and final section describes the stimulation operation in the field, offering a comprehensive account of injection rates, volumes, and pressures. This operational view distinguishes this study, as it covers all three essential stages concurrently, a feat not commonly seen in recent researches. Unlike previous studies that often neglected stages or focused only on oil wells, this approach thoroughly addresses each steps, providing a holistic understanding of the VDA system's application in gas wells.

The research emphasizes the unique properties of gas reservoirs and their impact on the effectiveness of VDA systems compared to oil wells. The findings highlight the distinct outcomes achievable with the VDA system in gas wells, contributing significantly to the understanding of reservoir stimulation in these specific contexts.

Furthermore, the paper unveils specific details about the VDA composition, a level of disclosure not commonly found in similar investigations. The disclosed information includes the percentages of Hydrochloric Acid (87.8%), Corrosion Inhibitor (1.2%), Corrosion Inhibitor Intensifier (0.8%), Non-Emulsifier (0.7%), Iron Sequestering Agent (1%), Methanol (1%), and Visco-Elastic Surfactant (7.5%). This transparency provides a unique insight into the operational specifics of the acidizing approach, setting this study apart from others in the field.

In conclusion, comprehensive approach, detailed composition disclosure, and emphasis on gas well characteristics make this study a significant advancement in the field of reservoir stimulation.

## Materials

This section describes the materials and additives utilized in this project's acid treatment. The rock mineralogy, reservoir temperature, permeability, and compatibility between the additives and the in-situ fluid all played a role in the choice of acid additives. The pre-flush and over-flush stages utilized the following additives: surfactant, mutual solvent, methanol, isopropanol, and H_2_S scavenger. In addition to the previously mentioned substances, the main acid stage contained a corrosion inhibitor, a corrosion inhibitor intensifier, a non-emulsifier, an iron sequestering agent, and an iron chelating agent. The main acid for this stage was 28% HCl. Also, a visco-elastic surfactant was applied in the VDA stage to enhance the acidizing performance.

## Methods

### Laboratory tests

#### Dissolution test

Before acidizing operations, a laboratory acid dissolution test is conducted to determine the reaction of the acid with the formation rock. The acid dissolution test can help optimize the acidizing design by identifying the optimal acid type, concentration, and volume for a specific formation. The acid dissolution test can also be used to determine the amount of dissolved rock, the change in permeability, porosity, or wettability, and the presence of insoluble residues or particles. The acid dissolution test involves exposing a rock sample to an acid solution under simulated reservoir conditions and measuring the weight loss, surface area, and pore volume of the sample before and after the test. The acid dissolution test can also aid in preventing formation damage, acid leakage, and insufficient coverage, all of which can reduce the acidizing's effectiveness or lead to operational complications^37,38^.

As part of the investigation, acid dissolution tests were conducted to determine how the rock reacted to different acid systems. In the experimental design, emphasis was placed on optimizing the concentration of hydrochloric acid. To achieve this, various concentrations of hydrochloric acid were added to the acid solution, namely 7.5%, 15%, and 28%. A 30-g rock sample composed of carbonate, dolomite, and a typical reservoir rock was selected for the dissolution experiments. The procedure for dissolution was initiated by immersing the rock sample in 150 mL of an acid solution. To determine the optimal acid concentration, weight concentrations of 7.5%, 15%, and 28% were compared in order to identify the concentration with the highest acid dissolution rate. Under two surface temperatures of 194°F, a series of six-hour-long tests were conducted to determine the optimal acid concentration. During these experiments, the acid concentrations changed, and the additives Corrosion inhibitor, Corrosion Inhibitor Intensifier, Non-Emulsifier, Iron Sequestering Agent, Iron Chelating Agent, Surfactant, and H_2_S Scavenger were added to determine their impact on the dissolution procedure. The experimental method permitted the comparison and evaluation of the solubility response of the rock sample to varying acid concentrations and the influence of additives at controlled temperatures. This analysis helped determine the optimal acid concentration for subsequent acidizing operations by providing valuable insight into the solubility behavior of the rock and its interaction with the acid systems tested. In this study, acid solubility tests were performed on both the main acid and the Viscoelastic Diverting Agent (VDA).

#### Emulsion test

The Emulsion Test is a laboratory test that is performed before acidizing operations to ensure that the acid and its additives are compatible with the crude oil (condensate) in the formation. Emulsion testing can help prevent potential problems such as emulsion formation, sediment formation, and oil viscosity increases, which can reduce the acidizing process's effectiveness or cause operational complications. The emulsion test entails combining the acid solution with the sample of the condensate under conditions simulating reservoir conditions and observing the resulting reactions and changes^9,39^.

In this experiment, a 100-ml cylinder is filled with 2.5 g of a sieved mixture of 90 percent silica flour and 10 percent bentonite powder. Then, 50 ml of hydrochloric acid, VDA, a predetermined amount of anti-emulsion additive, and other additives are added to the cylinder. The mixture is then supplemented with 500 ppm of Fe^3+^ ions from FeCl_3_ salt and 50 ml of well condensate. The contents of the cylinder are then stirred at 14,000 revolutions per minute for 30 s. The cylindrical object is then placed in an oven set to 194 degrees Fahrenheit. For thirty minutes, phase separation is measured and reported at 5-min intervals. The optimal anti-emulsion additive concentration is defined as the minimum concentration required to completely separate the two phases within 30 min. The test is repeated using spent acid, and the results are reported. In addition, the maximum acceptable anti-emulsion additive concentration is restricted to 3% by volume.

#### Iron control test

Iron precipitation is a common issue during acidizing because it reduces the permeability of the well. Iron (III) deposits a black precipitate when the pH exceeds 2, and iron (II) produces iron (II) carbonate when it reacts with calcium carbonate over time. This can be avoided by adding an iron control agent to the acid solution^40^. The effectiveness of the iron-control agent can be evaluated in the following ways: First, 100 ml of iron chloride (FeCl_3_) and acid are combined to produce a solution with a specified concentration of iron (III) ions (500 ppm or 1000 ppm)^9^. After the addition of calcium carbonate, which raises the solution's pH to 2, a diluted sodium hydroxide solution raises the pH to 4. If there is no red iron (III) hydroxide (Fe(OH)_3_) precipitate, the solution is baked at 194 degrees Fahrenheit for six hours. The concentration of the iron control agent is recorded after the test to determine if the solution is clear and free of particles. In the absence of a positive result, the test is repeated with a higher dose of the agent. The optimal dose of the agent is the lowest concentration necessary to maintain the solubility of iron ions throughout the test.

#### Corrosion test

Acidizing can corrode the pipes and equipment used in oil and gas operations as well as pose environmental and safety risks. Consequently, acidizing requires proper planning, design, and implementation^41^. Corrosion inhibitors are chemicals that protect metal surfaces from acid attacks. They form a protective layer on the metal that reduces the rate of corrosion. Corrosion inhibitors are essential for acidizing operations because they prevent the deterioration of drill pipes, tubing, and wellhead lines. Corrosion inhibitors have limited effectiveness and can degrade over time or when exposed to high temperatures. Consequently, acidizing operations require corrosion monitoring^42–45^.

**Figure 1:**
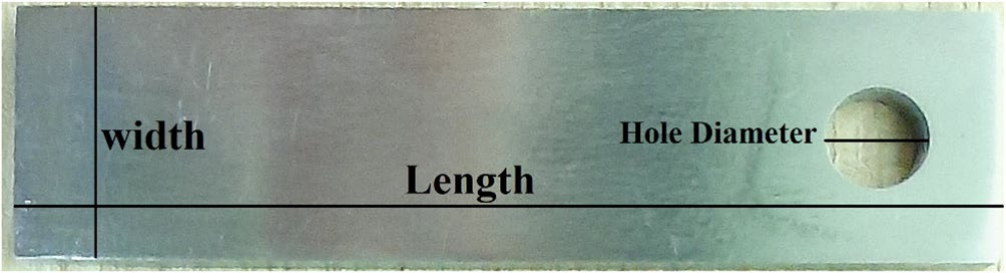
Corrosion coupon.

The method of weight loss was used to assess the corrosion of all acid solutions. Before the test, the dimensions (length, width, and height) of the corrosion coupon were measured and recorded (Table 1 and Fig. 1). The coupon was then immersed in a corrosion cell containing 500 ml of an acid solution with a corrosion inhibitor and corrosion inhibitor intensifier. Six hours were spent heating the cell at 194 degrees Fahrenheit in an oven. This procedure was performed on both acid solutions (Main Acid and VDA). After the dynamic thermal aging test, the coupon was remeasured and inspected for signs of corrosion, including holes, blisters, and other forms of corrosion. The corrosion rate (CR) was determined using Eq. 1^46,47^. The equation presented in numerous standards to calculate the CR, which is also widely employed in academic literature, is as follows:

1 $$CR=\frac{8.76\times {10}^{4}\times {W}_{loss}}{\rho At}$$

where CR is in [mm/y], W_loss_ is the weight loss in time t [hours], A is the surface area of the exposed corrosion coupon in cm^2^, and $\rho$ is the density of the corrosion coupon in g/cm^3^.

**Table 1:** Dimensions and weight of corrosion coupons before corrosion test.

	Hole Diameter (in)	Length (in)	Width (in)	Thickness (in)	Coupon initial weight (gr)
Main Acid	0.24	2.559	0.0866	0.00787	22.1779
VDA	0.24	2.559	0.0866	0.00787	22.1723

#### H_2_S scavenging test

Hydrogen sulfide (H_2_S) is a major problem in oil and gas operations, as it can be generated during acidizing treatments of oil and gas wells^48,49^. H_2_S is a toxic and corrosive gas that increases corrosion and iron sulfide formation, resulting in increased operating costs and decreased revenue. The gas is also a serious environmental and health hazard, particularly when it is produced when the acidizing treatment is reversed. This significantly increases the risk of stress corrosion cracking for any susceptible materials present in the well. Therefore, it is important to control and minimize the H_2_S emissions during and after the acidizing treatment. Utilizing H_2_S scavengers, which are chemicals that react with H_2_S and convert it into less hazardous compounds, is one method for reducing H_2_S emissions. H_2_S scavengers can be added to the acidizing fluids or injected separately before or after the acidizing treatment. However, not all H_2_S scavengers are equally effective and compatible with the acidizing fluids and the formation. Therefore, it is necessary to evaluate the performance of various H_2_S scavengers under varying conditions and choose the most appropriate one for the specific acidizing operation^50,51^.

**Figure 2:**
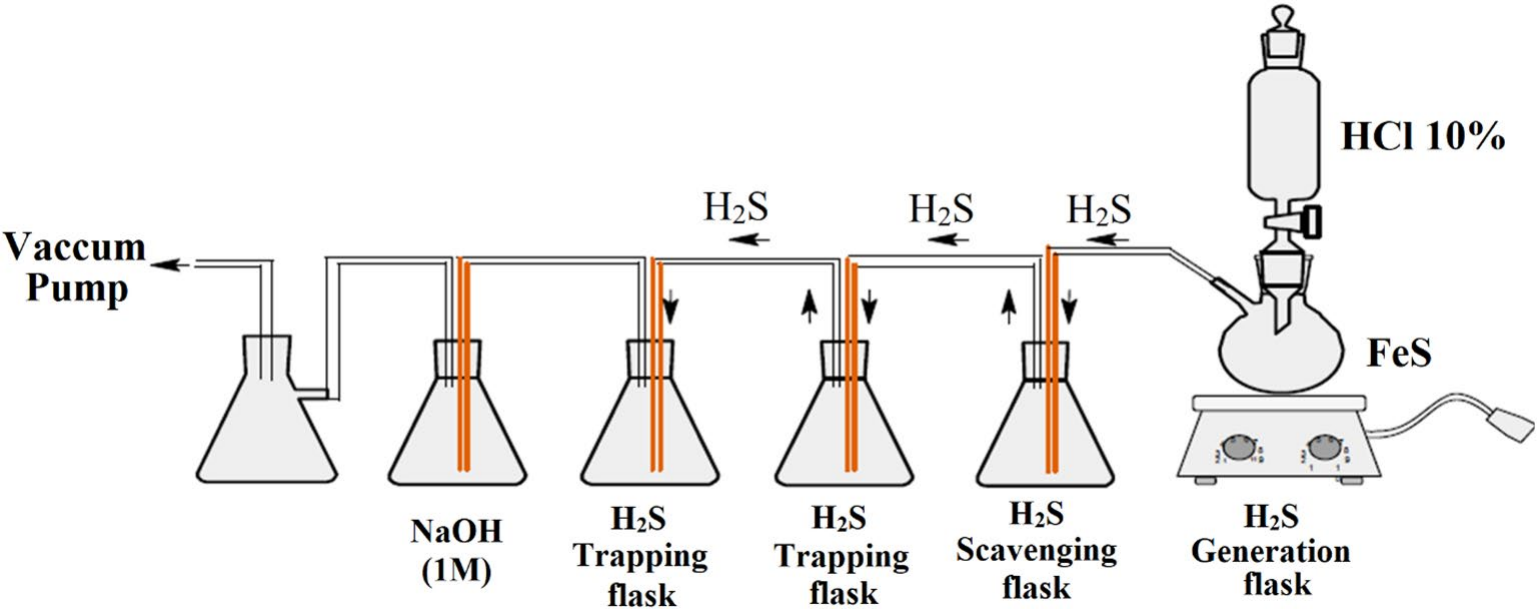
Experimental set-up of H_2_S scavenging test.

To evaluate the performance of H_2_S scavengers for matrix-acidizing treatment, the method of Xiao et al. was adopted^50^. This method generates H_2_S by reacting HCl acid with FeS, which is a common sulfide scale in oil and gas wells. The experimental setup was prepared as shown in Fig. 2. 10% HCl acid was poured into the dropping funnel and 1g FeS was placed into the two-necked flask, which had a hose to transfer the gas produced from the reaction.

$${\text{FeS}} + 2{\text{HCl}} \to {\text{FeCl}}_{2} + {\text{H}}_{2} {\text{S}}$$

The H_2_S from the reaction entered a H_2_S Scavenging flask that contained various solutions. The H_2_S Trapping flask contained 5 wt% CdSO_4_ solution as an absorbent. The amount of H_2_S generated was estimated from the amount of FeS reacted using stoichiometric equations. The residual H_2_S passed through the Scavenging flask and arrived at the Trapping flask, where it reacted with CdSO4 and formed a yellow precipitate of CdS as follows.

$${\text{H}}_{2} {\text{S}} + {\text{CdSO}}_{4} \to {\text{CdS}} \downarrow + {\text{H}}_{2} {\text{SO}}_{4}$$

The CdS precipitates were filtered using filter paper, dried in an oven at 150°C and measured using a high precision balance. For safety purposes, a flask containing 1M NaOH solution was put at the end of the system to neutralize any H_2_S escaping from the reaction system. The reactor and the absorbers were stirred while purging them with a vacuum pump. The amount of FeS, 10% HCl, CdSO_4_ solution and NaOH solution was fixed for all tests. However, the composition of the H_2_S Scavenging flask was changed for each test (0.5 to 1). To study the effect of temperature on the scavenger reaction, the H_2_S Scavenging flask was heated to 90ºC in a water bath. To study the effect of pH on the scavenger reaction, CaCO_3_ was added to 10% HCl to adjust the pH (pH = 4.5).

### Simulation

In this section, a detailed simulation was performed before the acidizing operation using Stimpro 2019 software (Version (10.11.61.0)). This simulation process was extremely beneficial for the design of injection stages, the determination of injection rate and pressure, and the subsequent analysis of operation results. The utilization of operation simulation led to a greater comprehension of critical factors and an acidizing operation that was optimized. The simulation model was developed by incorporating essential information and relevant inputs, resulting in a reliable and accurate representation of the acidizing operation. The simulation model incorporates the following key components:

**Well data** The drilling plan was used to obtain vital well data, including drill pipe and casing depths. These data components are shown in.

Table 2, which contains essential simulation parameters. The simulation software used these data as inputs to model the acidizing process.

**Table 2:** Well data for simulation.

SegmentType	Length (ft)	Pipe ID (in)	Pipe OD (in)	Weight (lb/ft)	Grade
Casing	573	24.750	26.000	169.380	L-80
Casing	3209	17.655	18.625	96.500	K-55
Casing	5087	12.415	13.375	68.000	L-80
Casing	10,783	8.680	9.625	47.200	L-80
Casing	1514	6.184	7.000	29.000	L-80
Tubing	10,330	6.366	7.000	22.640	L-80

**Reservoir data** Reservoir data provided information on essential reservoir layer properties, such as pressure, temperature, rock type, porosity, permeability, fracture pressures, and more. It should be mentioned that the initial skin of the reservoir is derived from the pressure transient analysis (well test). These properties are essential for designing and evaluating the acidizing operation. Table 3 is a summary of the reservoir data used for this study, compiled from field reports and published materials. The reservoir data were used as inputs by the simulation software to model the acid-rock reaction and wormhole propagation.

**Table 3:** Reservoir data for simulation.

Reservoir temperature (°F)	194	Average reservoir pressure (psig)	3612
Absolute Permeability (mD)	2.58	Porosity	0.108
Gas Viscosity at Reservoir Condition (cp)	0.030	Frac Pressure (psig)	6388
TVD to Top of Open Section (ft)	8771	TVD to Bot of Open Section (ft)	9483
Acidizing Type	Dolomite & Limestone	Acid Volume (bbl)	1715
Avg. Surface Pressure (psig)	875	Max. Surface Pressure (psig)	1604
Initial Skin	5		

**Skin value** The field-analyzed data helped determine the incorporation of skin value, a significant simulation-influencing parameter.

**Maximum surface injection pressure (MSIP)** Field-collected fracture pressure gradient data helped to determine the MSIP. This parameter ensures acidizing occurs below the fracturing pressure, a crucial safety factor.

**Treatment chemicals** A comprehensive evaluation of treatment chemicals, including preflush, main flush, VDA, and overflush, was conducted using laboratory tests, mathematical calculations, and historical field experience. Concentrations and volumes were taken into account.

**Diversion and pumping strategy** Guidelines and historical field experience were used to establish diversion and pumping strategies, thereby increasing the simulation's precision. A comprehensive treatment plan was developed after laboratory tests identified the chemical components. The schedule detailed the preflush, main flush, VDA, overflush, and displacement phases of the pumping procedure. Pumping rates and chemical volumes were meticulously included. During matrix acidizing, it was essential to keep the pumping pressure below the fracture pressure in order to preserve the matrix's integrity and prevent fractures. By effectively monitoring pumping pressure and rate trends, the simulator permitted treatment schedule modifications if the simulated pressure approached or exceeded the fracture pressure threshold.

### Operation

#### Carbonate acidizing

Carbonate acidizing is a method for increasing the productivity of carbonate reservoirs by injecting acid to create wormholes between the wellbore and oil or gas zones. Carbonate acidizing is a complex process that depends on many factors such as fluid flow, reaction kinetics, mass transfer, and rock heterogeneity^52^. Typically, the following steps are followed to achieve a successful carbonate acidizing treatment^11^:

- **Cleaning the wellbore** This step entails pumping 10–15% HCl acid with additives to remove scale, rust, and deposits from the tubing and vicinity of the wellbore.
- **Pre-flushing the formation** Formation Pre-flushing entails pumping a fluid that prepares the formation for the main acid stage. The pre-flush fluid acts as a separator between the formation fluid and the main acid and also improves the rock's wettability to water, thereby enhancing the acid-rock reaction.
- **Injecting the main acid** In this step, the main acid that dissolves the carbonate rock and creates wormholes is injected. The principal acid should be compatible with the formation, gas, temperature, and additives.
- **Diverting the acid** Acid diversion entails the use of mechanical or chemical means to direct the acid to various zones or intervals of the formation. It is important because carbonate formations are typically heterogeneous and have high reaction rates, which can result in preferential acid flow paths. Diversion ensures that the acid is distributed uniformly along the wellbore.
- **Over-flushing the formation** Over-flushing the formation entails pumping a fluid that removes the acid and any insoluble reaction products from the wellbore. Additionally, the over-flush fluid aids in the removal of any blocking material that may have been produced in previous stages.
- **Displacement of fluids** This step involves pumping a fluid that displaces all of the over-flush fluid into the formation and facilitates the flow of fluids back into the reservoir. This step is particularly crucial for low-pressure reservoirs.

#### Acidizing parameters

The following parameters must be determined in order to design an effective acidizing treatment for carbonate reservoirs: acid volume, injection rate, and injection pressure. These parameters are dependent on the desired wormhole penetration and the conditions of the reservoir. The following are the methods for calculating these parameters:

**Acid volume** This parameter specifies the volume of acid required to create wormholes with a given radius. For estimating acid volume per unit thickness of formation, there are two methods: (1) Daccord's wormhole propagation model, which is based on the fractal geometry of wormholes, and (2) the volumetric model, which is based on the pore volume of acid injected at the wormhole breakthrough^53^. The first method is optimistic in that it assumes uniform acid distribution, while the second method is more realistic and requires laboratory data^53^.

**Injection rate** This parameter represents the acid injection rate into the formation. However, this may necessitate a larger acid volume. Consequently, the injection rate must be optimized based on the available acid volume and the reservoir's characteristics. For limestone formations, the maximum injection rate is recommended, whereas for dolomites, a reduced injection rate may be preferred to increase acid temperature and reaction rate.

**Injection pressure** This parameter specifies the pressure required to inject the acid at the specified rate. It is possible to measure the injection pressure at the surface or in the borehole.

#### Operation procedure

**Figure 3:**
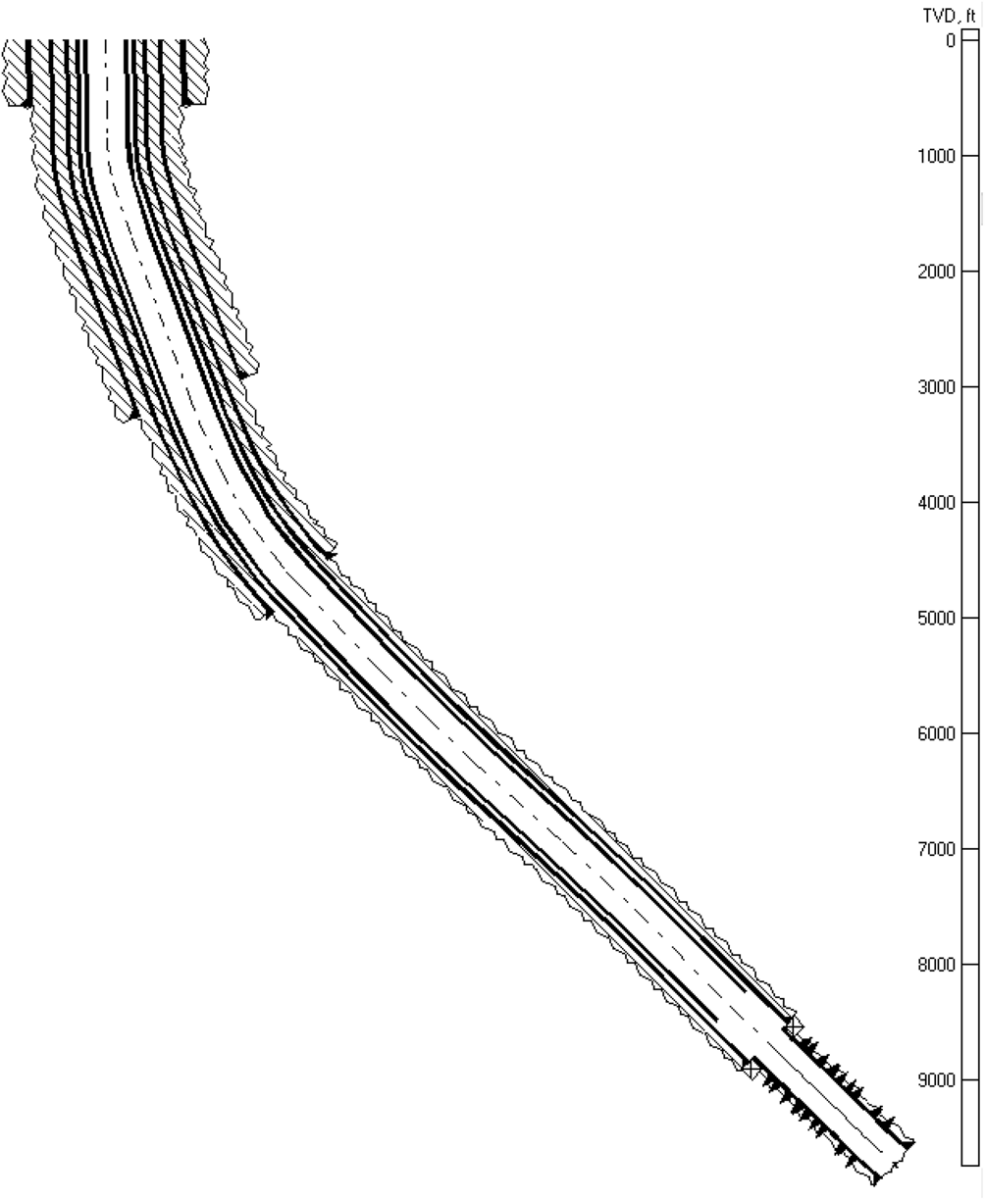
Wellbore schematic.

This project was carried out on a gas well in a southern Iranian gas field. The reservoir consists of three dolomite-dominated layers. The reservoir was chosen for acid treatment in order to increase its gas output. The characteristics of the well and reservoir are shown in Table 4, and the schematic of the well is depicted in Fig. 3.

**Table 4:** Well Data.

FORMATION	Layers A, B, C
RESERVOIR ROCK TYPE	Dolomite & Limestone
H_2_S	1800 ppm
CO_2_	1%
TOTAL INTERVAL	518.4 ft
RESERVOIR PRESSURE	A: 4300 psi B: 3200 psi Top of C: 3200 psi, Bottom of C: 4000 psi
FRACTURE GRADIENT	0.7 psi/ft
GAS GRADIENT	0.117 psi/ft
Well HEAD PRESSURE	1075 psi
FLOW RATE	26 MMSCF/D
SHOT/ft (SPF)	6 S/F
ACID VOLUME/ft	70 gal/ft
STAGE INTERVAL	100 ft/interval

**Figure 4:**
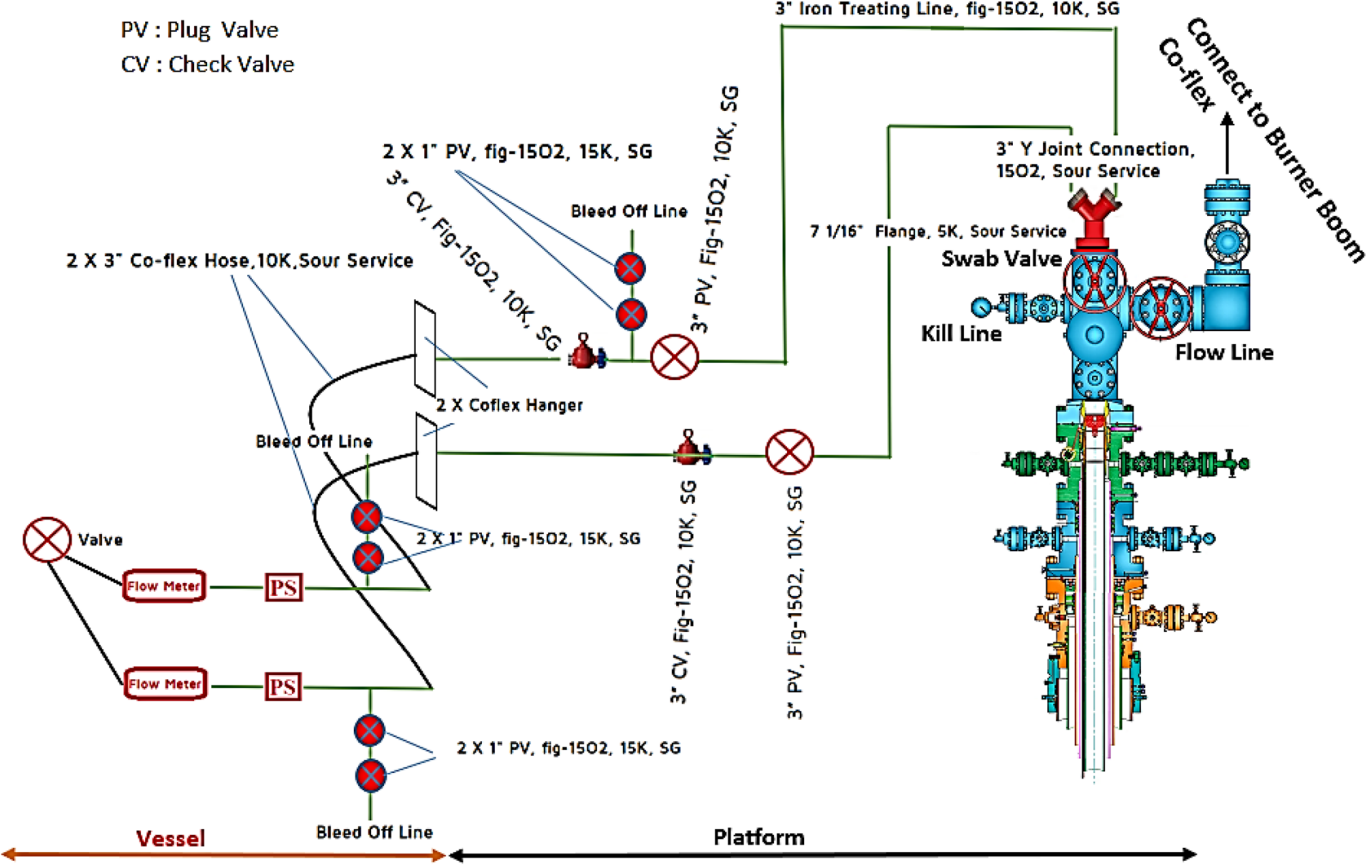
Schematic of how to connect the ship to the wellhead.

An acid vessel was used for the acid treatment. The acid composition was determined based on the outcomes of the rock and fluid tests and the acid simulation. The acid was prepared in special tanks on the vessel and then pumped into the reservoir using high-pressure pumps on the vessel and lines connected to the wellhead, as per the acidizing program. Figure 4 depicts the vessel and well's connection scheme.

**Figure 5:**
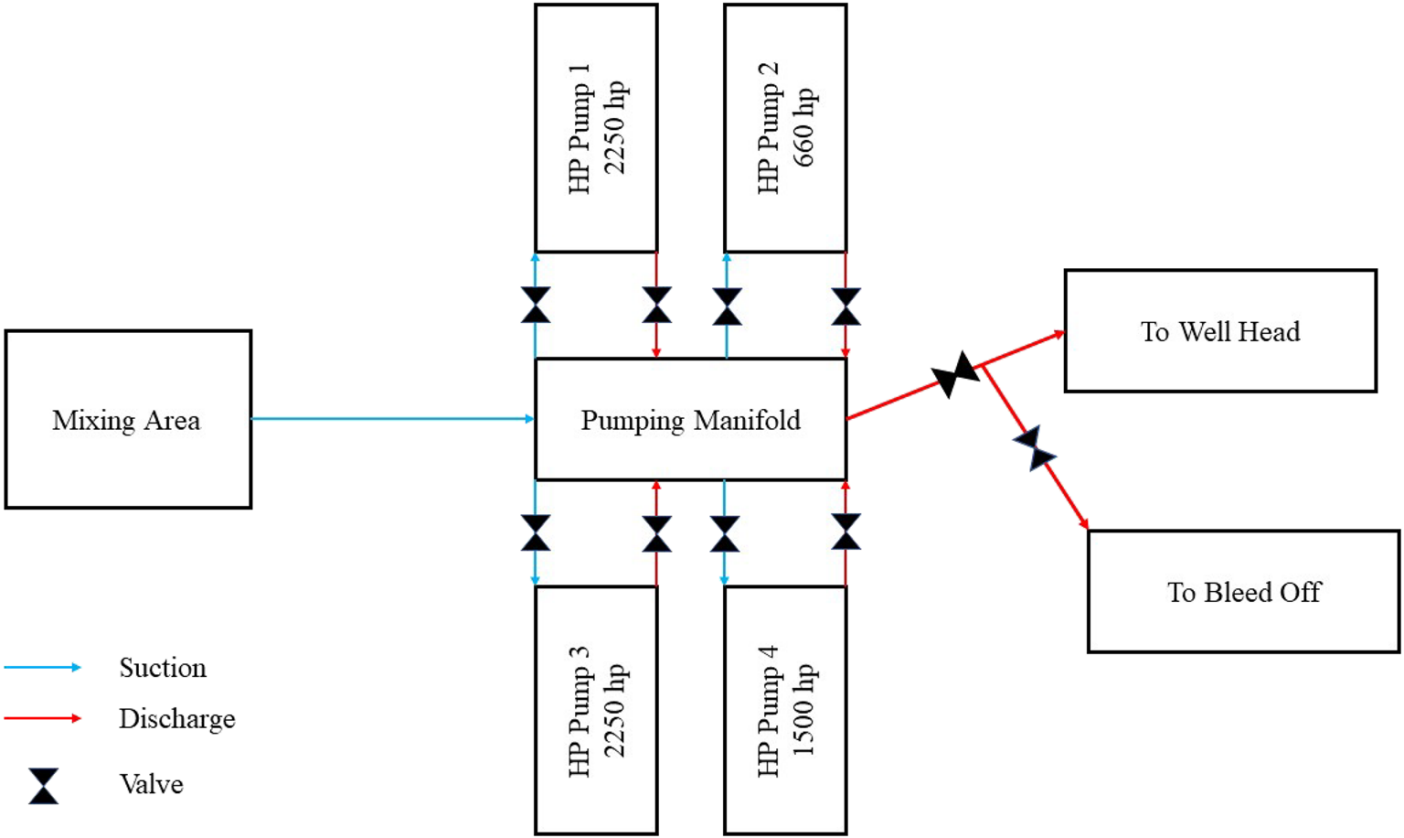
Schematic of the connection between different pumps.

In this study, four high-pressure pump units connected in parallel have been utilized for acid injection. These pumps include two 2250 hp pumps, one 1500 hp pump, and one 660 hp pump. Consequently, with the assistance of this equipment, the desired injection rate can be easily achieved. The schematic of the connection between different pumps is illustrated in the Fig. 5.

The acidizing procedure consisted of the following steps:

1. Connect the vessel lines to the wellhead.
2. Check the line pressure to ensure that there are no leaks at the connections.
3. According to the simulation results, mix and pump the pre-flush, main acid, diverter, and over-flush fluids.
4. Replace and flush the acid with treated water and then salt water up to 100 feet above the A layer.
5. Disconnect the stimulation lines from the rig/platform and secure the well.

## Results and discussion

### Laboratory tests

#### Main acid

##### Dissolution test

The acid dissolution study incorporated various concentrations of hydrochloric acid (7.5%, 15%, and 28%), constituting the acid solution integral to the investigative approach. Table 5 presents a comprehensive list of additives included in the solution, providing a detailed overview of the experimental composition.

**Table 5:** List of ingredients in acid solution.

Contract description	Quantity (%)
Hydrochloric Acid	89.36
Corrosion Inhibitor	1.43
Corrosion Inhibitor Intensifier	0.95
Non-Emulsifier	0.67
Iron Sequestering Agent	0.93
Iron Chelating Agent	4.76
Surfactant	0.95
H_2_S Scavenger	0.95

**Figure 6:**
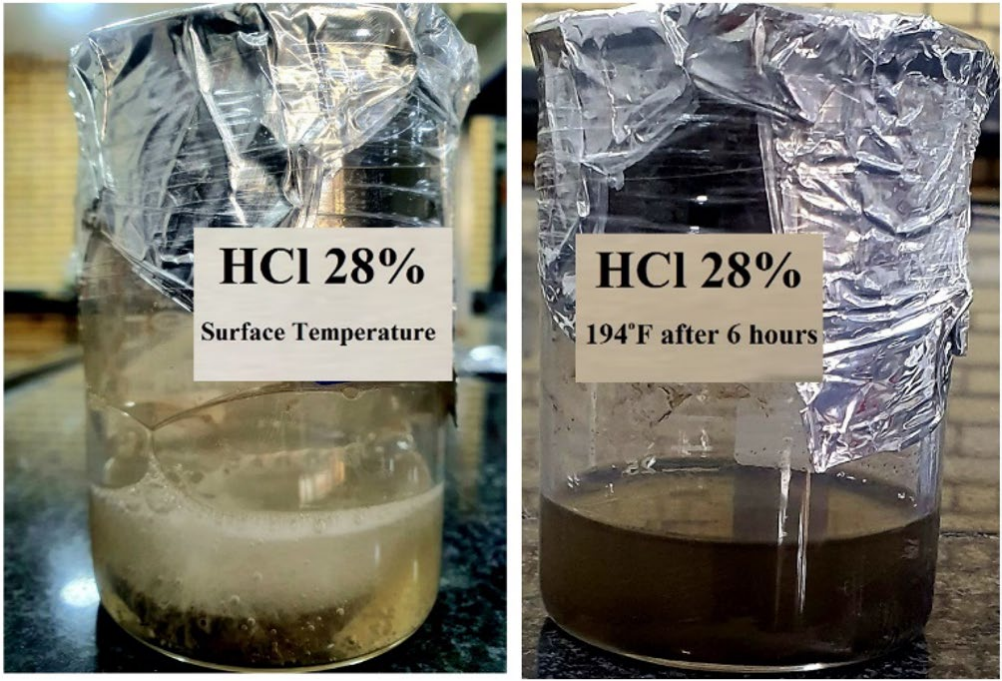
Results of the acid dissolution.

Figure 6 functions as a visual representation of the acid dissolution process, effectively illustrating the outcomes of the investigation. Notably, the application of a 28% hydrochloric acid concentration revealed remarkable results, demonstrating the highest percentage of dissolution and unparalleled overall performance. This concentration proved highly effective in nearly complete limestone sample dissolution at both investigated temperatures. The findings highlight the superiority of the 28% hydrochloric acid concentration, positioning it as the optimal choice for acidizing carbonate and dolomite reservoir materials. The substantial dissolution observed implies a potent capability to degrade limestone samples efficiently, signifying its potential for robust and effective acidizing operations in real-world field applications.

##### Emulsion test

The part presents the results of emulsion tests examining the impact of anti-emulsion additive on the separation of condensate and main acid mixtures into separate phases, using three different ratios of condensate and main acid (25:75, 50:50, and 75:25) and testing live and spent main acid to simulate different acidizing stages.

The results of the tests showed that the anti-emulsion additive worked in all cases to stop emulsions from forming and improve phase separation. For live main acid, no emulsion was observed in any of the tests, regardless of the ratio of condensate and main acid or the concentration of the additive. The complete phase separation occurred in less than 6 min for all conditions. For spent main acid, no emulsion was observed in any of the tests, and the complete phase separation occurred in less than 3 min for all conditions. The optimal concentration of the anti-emulsion additive was identified as 0.67% for both live and spent main acids, as lower concentrations led to incomplete phase separation within 30 min. These outcomes show that the anti-emulsion additive can effectively eliminate emulsion problems and improve fluid placement in acidizing operations. The additive can be applied in various ratios of condensate and main acid, both live and spent, without compromising its performance. The additive can also reduce operational costs and environmental impacts by minimizing the amount of chemicals needed to achieve optimal phase separation. Therefore, the use of this additive is recommended as a beneficial solution for acidizing operations in carbonate reservoirs.

##### Iron control test

**Figure 7:**
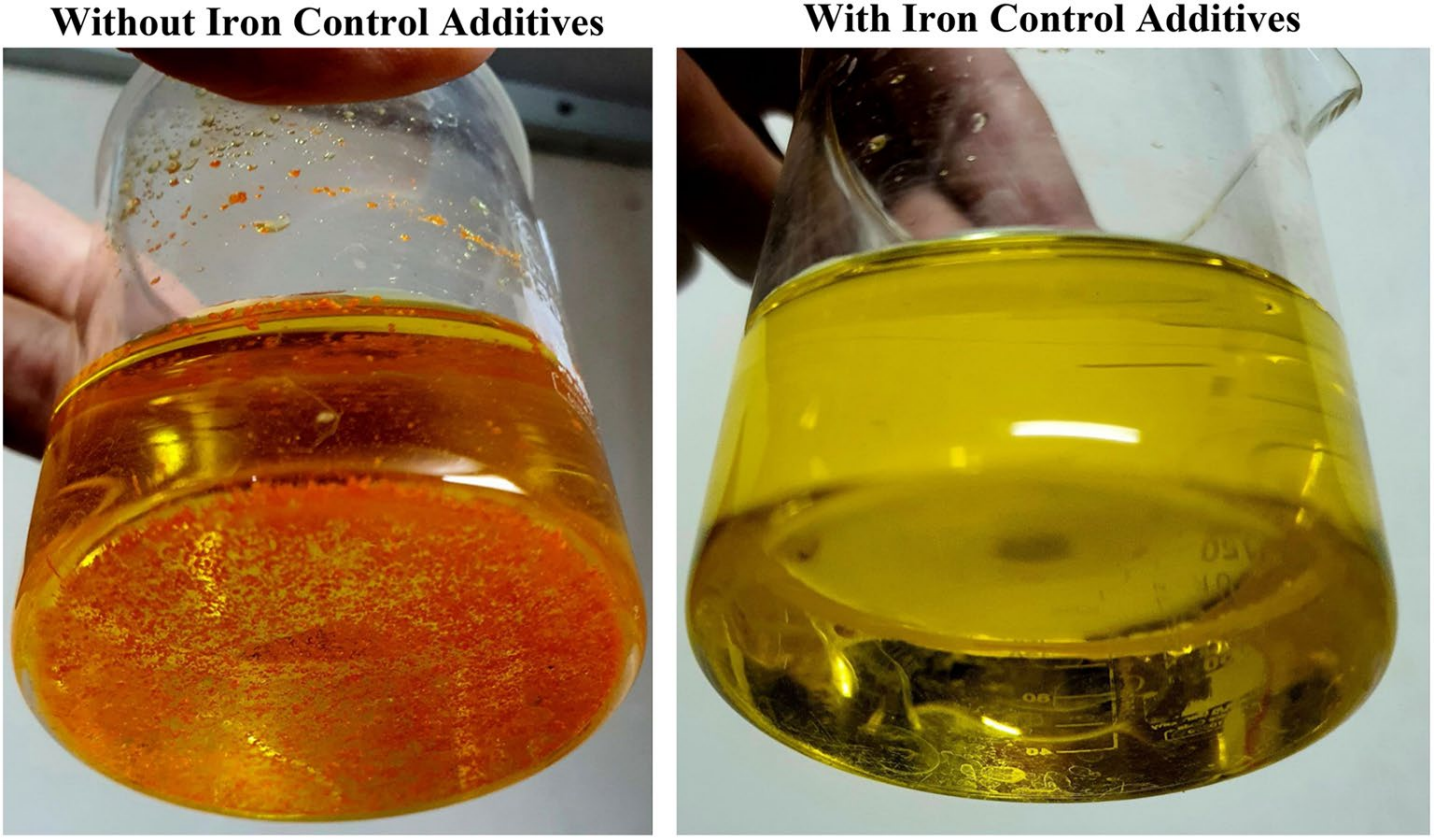
Iron control additives.

The Iron Control Test was conducted to evaluate the efficiency of Iron Control additives in preventing iron precipitation. Figure 7 illustrates the images of the acid solutions following the test with and without Iron Control.

The effect of varying the concentrations of the Iron Sequestering Agent and the Iron Chelating Agent (from 0.5 to 0.93 for the former and 3 to 4.76 for the latter) was investigated. The results indicated that 0.93 percent by volume of Iron Sequestering Agent and 4.76 percent by volume of Iron Chelating Agent were the optimal concentrations for these two additives, as they maintained the solubility of the iron ions throughout the test.

##### Corrosion test

In this section, a corrosion test was conducted to evaluate the effectiveness of corrosion inhibitors and corrosion inhibitor intensifiers on the corrosion rate of the main acid. This test was initially conducted with the percentages recommended by the manufacturer of the corrosion inhibitor and corrosion inhibitor intensifier. The corrosion rate must be less than 45 mm per year over a period of six days. Table 6 displays the input parameters of Eq. 1, including weight loss, time, surface area, corrosion sample density, and corrosion rate. Using this equation, the precise corrosion rate was calculated.

**Table 6:** Corrosion results for the main acid.

Coupon weight after test (gr)	W_loss_ (gr)	$\rho$(pcf)	A (ft^2^)	T (hr)	CR (mm/y)
21.8349	0.343	504.0433	0.015	6	44.25

The results show that the addition of 1.43% and 0.95% by volume of corrosion inhibitor and Corrosion Inhibitor Intensifier, respectively, decreased the corrosion rate of the main acid to about 44.25 mm per year. Although this value is close to the permissible limit of 45 mm per year, it is still acceptable. Noting that acid inhibitors are typically highly concentrated compounds, reducing the quantity of corrosion inhibitors can help acidizing operations save money. Consequently, the results suggest that a 1.43% addition of corrosion inhibitor and a 0.95% addition of Corrosion Inhibitor Intensifier can be a cost-effective method for reducing corrosion in acidizing operations.

##### H_2_S Scavenging test

**Figure 8:**
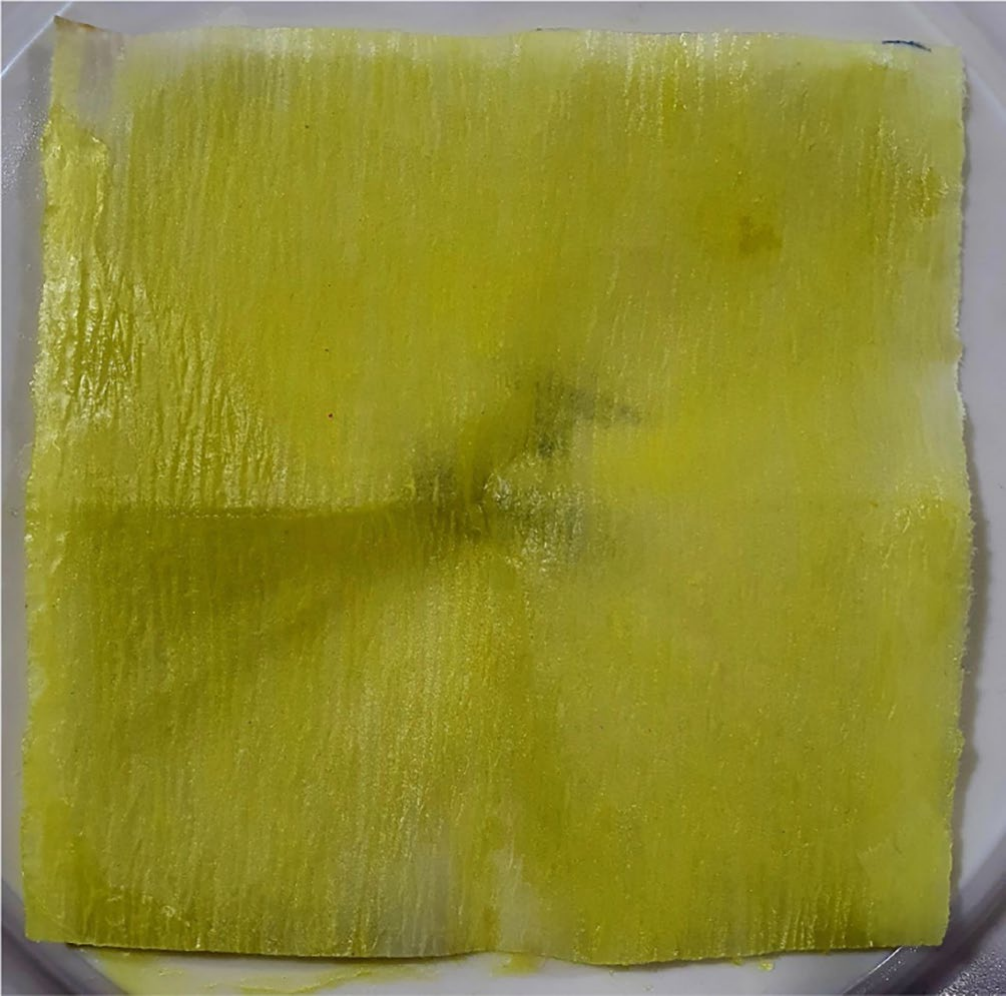
Precipitate of CdS @ trapping cylinder.

In this section, the effect of H_2_S scavenger addition is investigated. The optimal concentration of H_2_S scavenger was determined by measuring the quantity of CdS precipitate formed in the trapping flask after FeS and HCl reacted to produce H_2_S gas. The quantity of CdS precipitate at various H_2_S scavenger concentrations (0.5 to 1 wt%) was measured. As the concentration of H_2_S scavenger went up, it was seen that the amount of CdS precipitate went down. This shows that the solution took in more H_2_S gas. The concentration of 0.95 wt% H_2_S scavenger that produced the smallest quantity of CdS precipitate (0.02 g) was chosen as the optimal concentration for further testing. The appearance of the filter paper after passing through a 0.95 wt% H_2_S scavenger solution is depicted in Fig. 8. It was observed that the filter paper had a negligible quantity of yellow CdS deposits, confirming that the solution captured the majority of the H_2_S gas.

The results of this study demonstrate that H_2_S scavengers can effectively reduce the amount of H_2_S gas generated from FeS and HCl reactions in matrix-acidizing treatments. The optimal concentration of H_2_S scavenger was determined to be 0.95 weight percent, which resulted in the lowest amount of CdS precipitate in the trapping cylinder.

#### Viscoelastic diverting acid (VDA)

##### Dissolution test

In this section, the VDA Dissolution Test was carried out to assess the performance of VDA in disintegrating carbonate and dolomite reservoir materials. Utilizing VDA, a specific formulation was created to facilitate the dissolution process. Different concentrations of hydrochloric acid (7.5%, 15%, and 28%) were used to formulate the study's solution. In addition to hydrochloric acid, the solution contained the additives detailed in Table 7.

**Table 7:** List of ingredients in VDA solution.

Contract description	Quantity (%)
Hydrochloric Acid	87.8
Corrosion Inhibitor	1.2
Corrosion Inhibitor Intensifier	0.8
Non-Emulsifier	0.7
Iron Sequestering Agent	1
Methanol	1
Visco-Elastic Surfactant	7.5

Observations were made during the Live VDA solution test that the acid had not yet reacted with the rock sample. The result was that the solution remained liquid and was soluble at both temperatures. This result indicates that when VDA is injected into a well, it first exists as a solution before dissolving the minerals. In contrast, the dissolution of the rock sample at the surface temperature conditions of the Spent VDA solution caused the entire solution to transition into a gel. After being exposed to a temperature of 194 degrees Fahrenheit for two hours, the solution reverted to a viscous liquid state. This phenomenon indicates that the VDA solution, after being injected into the formation and undergoing mineral dissolution, possesses viscosity. This viscosity permits the transient occlusion of highly permeable channels, increasing the acid's depth of penetration.

**Figure 9:**
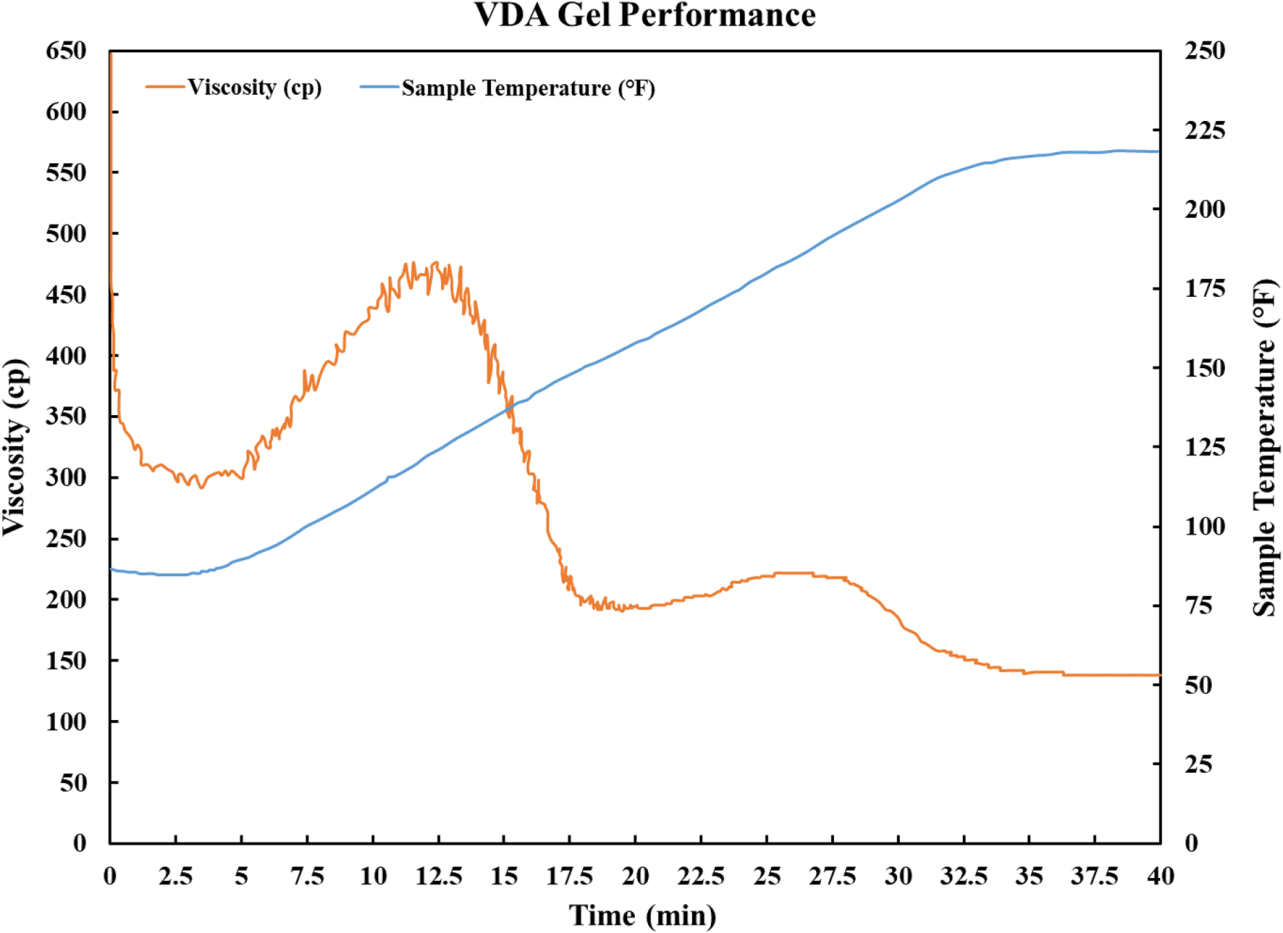
VDA gel performance.

One of the goals of this study was to assess the efficiency of VDA as a diverting agent for enhancing gas recovery. This was accomplished by measuring the viscosity of VDA at various temperatures and contact times with the reservoir rock. Figure 9 depicts the outcomes of the experiment.

Figure 9 illustrates the relationship between viscosity and temperature for VDA after its reaction with the rock. VDA demonstrates a thermo-reversible gelation behavior, meaning that its viscosity varies depending on the temperature. When VDA comes into contact with the rock at room temperature, it begins to form a gel-like structure. This results in increased viscosity and decreased mobility. After roughly 12.5 min, VDA reaches its maximum viscosity, which is approximately 460 cp. This shows that the VDA is capable of effectively plugging the high-permeability zones in the reservoir and redirecting the flow to the low-permeability zones. However, this gelation process is reversible; as the temperature rises, the gel structure begins to degrade and the viscosity of VDA decreases. This is desirable because it facilitates the removal of VDA from the reservoir once its diversion purpose has been satisfied. The minimum viscosity of VDA occurs at about 194 degrees Fahrenheit, which is close to the temperature of the reservoir. This indicates that VDA can recover its original mobility and flow towards the production well without incurring any damage or residual resistance. On the basis of these findings, it is possible to conclude that VDA is an effective agent for enhancing gas recovery in high-temperature reservoirs. It can produce a temporary and reversible plugging effect in high-permeability zones and enhance the sweep efficiency of injected fluids in low-permeability zones. In addition, due to its thermo-reversible gelation property, it poses a low risk of causing formation damage or reducing well productivity.

##### Emulsion test

The part examines the impact of anti-emulsion additive on the separation of condensate and VDA mixtures, using three different ratios (25:75, 50:50, and 75:25) and live and spent VDA.

The results of the tests showed that the anti-emulsion additive worked in all cases to stop emulsions from forming and improve phase separation. For live VDA, no emulsion was observed in any of the tests, regardless of the ratio of condensate and VDA or the concentration of the additive. The complete phase separation occurred in less than 6 min for the 25:75 ratio and less than 3 min for the 50:50 and 75:25 ratios. For spent VDA, no emulsion was observed in any of the tests, and the complete phase separation occurred in less than 6 min for the 25:75 ratio and less than 3 min for the 50:50 and 75:25 ratios. The optimal concentration of the anti-emulsion additive was identified as 0.7% for both live and spent VDA, as lower concentrations led to incomplete phase separation within 30 min.

##### Corrosion test

The purpose of this section was to examine the corrosion rate of the VDA in the presence of corrosion inhibitors and Corrosion Inhibitor intensifiers. The corrosion test was conducted at a temperature of 194 degrees Fahrenheit for six hours, and the manufacturer recommended that the corrosion rate be less than 45 mm per year.

Table 8 presents the input parameters used to calculate the precise corrosion rate using Eq. 1. The addition of 1.2% and 0.8% by volume of corrosion inhibitor and Corrosion Inhibitor Intensifier, respectively, resulted in a corrosion rate of approximately 5.88 mm per year. The corrosion results of the main acid and VDA indicate that VDA had much lower corrosion.

**Table 8:** Corrosion results for the VDA.

Coupon weight after test (gr)	W_loss_ (gr)	$\rho$(pcf)	A (ft^2^)	T (hr)	CR (mm/y)
22.1267	0.0456	503.9	0.015	6	5.88

### Simulation

#### Acidizing stages design

This section of the article discusses the acidizing operation using the designed solutions for the pre-flush and main acid stages. The pre-flush phase was conducted to remove brine from the wellbore and prepare the reservoir for the acid treatment's primary phase. The pre-flush solution contained a detergent, methyl alcohol, isopropyl alcohol, and an H_2_S scavenger. The surfactant was used to reduce the interfacial tension and alter the wettability of the rock surface from oil-wet to water-wet. This means that the surface of the rock is more likely to be wetted by water than by oil (condensate), facilitating the displacement of oil (condensate) by water and improving recovery efficiency. As solvents, methyl and isopropyl alcohol were used to remove the condensate coating and avoid water blockage. The H_2_S scavenger was utilized to decrease the amount of hydrogen sulfide that could react with iron to produce undesirable iron sulfide precipitates. The composition of the pre-flush solution is detailed in Table 9.

**Table 9:** Non-acidic pre-flush.

Contract description	Quantity (bbl)
Mixing Water	63.1
Surfactant	0.9
ISO Propanol	4.3
Methanol	8.6
Mutual Solvent	8.6
H_2_S Scavenger	0.9

To induce wormholes in the carbonate formation and avoid the damage zone, the main acid treatment phase was carried out. Based on laboratory tests, 28% HCl was selected as the main acid. As additives, the main acid solution included corrosion inhibitors, corrosion inhibitor intensifiers, non-emulsifiers, iron sequestering agents, iron chelating agents, surfactants, and H_2_S scavengers. The corrosion inhibitor and intensifier were used to protect the surface equipment and production pipes from acid corrosion. The non-emulsifier was used to prevent the formation of emulsions, which could have slowed the acid-rock reaction. The iron sequestering agent and chelating agent were used to regulate the pH of the solution and prevent iron precipitation that could clog the pores. The composition of the main acid solution is shown in Table 10.

**Table 10:** Main acid.

Contract description	Quantity (bbl)
Hydrochloric Acid	810.4
Corrosion Inhibitor	13.0
Corrosion Inhibitor Intensifier	8.6
Non-Emulsifier	6.0
Iron Sequestering Agent	8.4
Iron Chelating Agent	43.2
Surfactant	8.6
H_2_S Scavenger	8.6

Viscoelastic Diverting Acid is an acidizing fluid that moves on its own and doesn't contain polymers. It can be used alone or in addition to other acids to treat all carbonate reservoir zones. VDA thickens as it stimulates carbonate formations, diverting the remaining acid treatment fluid into zones with lower injectivity for enhanced zone coverage across long intervals and high permeability differences. VDA also demonstrates efficient wormholing behavior in a wide variety of conditions, more effective leak-off control than straight hydrochloric and non-cross-linked gelled acid, high fluid efficiency during acid treatments, and simple mixing for smaller equipment. The components of the VDA solution are shown in Table 11.

**Table 11:** VDA diverter.

Contract description	Quantity (bbl)
Hydrochloric Acid	758.6
Corrosion Inhibitor	10.4
Corrosion Inhibitor Intensifier	6.9
Non-Emulsifier	6.0
Iron Sequestering Agent	8.6
Methanol	8.6
Visco-Elastic Surfactant	64.8

The VDA solution included viscoelastic surfactant, a cationic additive that increases the viscosity of hydrochloric acid and imparts a linear flow to the acid mixture. This aids in controlling the placement and penetration of acid within the formation and prevents the deposition of insoluble particles within the pore space. The objective of the over-flush stage was to displace the main acid flush away from the wellbore and to push the precipitation products as far away from the critical region as possible. The over-flush solution contained water or brine, which assisted in minimizing the acid's contact time with the tubing and casing. Table 12 displays the constituents of the overflow solution.

**Table 12:** Over-flush.

Contract description	Quantity (bbl)
Mixing Water	117.0
Surfactant	1.5
Methanol	14.8
Mutual Solvent	14.8

Using VDA in the diversion phase necessitated the injection of diesel and a common solvent during the post-flush phase. This treatment helped dissolve any emulsions or gel blocks that may have formed as a result of the relatively high concentration of surfactant used in the formulation of the VDA. Additionally, the over-flush phase assisted in dissolving corrosion inhibitors that had accumulated on the formation and decreased its permeability.

#### Acidizing parameters

This study investigated the effect of acid volume and injection rate on the formation and propagation of wormholes in carbonate reservoirs. Based on the reservoir parameters and the desired wormhole characteristics, the acid volume and injection rate were calculated using the formulas described in the methodology section. The selected acid volume of 70 gal/ft was sufficient to create wormholes that could penetrate beyond the damaged zone and increase the formation's permeability. The injection rate was determined to be within the optimal range for generating stable, uniform wormholes. A formula was used to calculate the maximum surface injection pressure (MSIP) for matrix acidizing based on the reservoir depth, fracture gradient, and hydrostatic pressure gradient. The MSIP represents the maximum surface pressure that can be applied without fracturing the formation. For this project, the fracture gradient of the reservoir was approximately 0.7 psi/ft, and the maximum hydrostatic pressure gradient in the acidizing operation was about 0.49 psi/ft. For this project, a safety margin of 5 percent, or 200–500 psi, was assumed. Taking into account the well's true vertical depth (TVD) and measured depth (MD), the MSIP was 1841.72 psi.

#### Simulation output

The acidizing treatment for carbonate reservoirs was simulated using Stimpro software based on the calculated fracture pressure and treatment volume, as detailed in the methodology section. The fracture pressure and treatment volume were determined by the porosity, permeability, and damage radius of the reservoir. The acidizing treatment was designed to inject acid below the fracture pressure to prevent formation fracturing and to create wormholes that could bypass the damage zone and increase the well's productivity. Based on the results of skin reduction, the simulation tool optimized the number of stages and injection rate for each stage. The design also accounted for the field's historical experience. Table 13 presents a summary of the volume requirements for treatment.

**Table 13:** Treatment volume requirements.

Stage	Volume (gal/ft)	Total volume (bbl)
Non-Acidic Pre-Flush	7	86.4
28% HCl Main Acid	70	864.0
VDA Diverter-15% VDA Acid	70	864.0
Over flush	12	148.1
Displacement	34	420.0

The optimal number of stages and injection rate for each stage are calculated and displayed in Table 14 based on the well condition and simulation results.

**Table 14:** Optimum number of stages and injection rate.

Step	Step type	Elapse time (min:sec)	Fluid volume (bbl)	Flow rate (bpm)
1	Preflush	8:38	86.4	10
2	Main acid	20:09	172.8	15
3	Diversion	28:47	216.0	25
4	Main acid	33:44	172.8	35
5	Diversion	39:08	216.0	40
6	Main acid	43:27	172.8	40
7	Diversion	48:51	216.0	40
8	Main acid	53:10	172.8	40
9	Diversion	58:34	216.0	40
10	Main acid	62:53	172.8	40
11	Overflush	66:11	148.1	45
12	Displacement	75:31	420.0	45

The results of the matrix acidizing simulation showed that the treatment was successful in creating wormholes that enhanced the permeability of the formation. Maximum surface injection pressure (MSIP) for matrix acidizing was calculated to be 1841.72 psi based on reservoir depth, fracture gradient, and hydrostatic pressure gradient using the formula described in the methodology section. The MSIP represents the maximum surface pressure that can be applied without fracturing the formation. This project required a treatment volume of approximately 70 gal/ft, which was sufficient to create wormholes that could penetrate beyond the damaged zone and increase the formation's permeability.

As shown in Table 13, the treatment fluid was simulated as a five-stage pumping operation. The pumping activity was maintained below the fracture pressure in order to prevent acid from moving preferentially into the fracture and leaving the unfractured zones untreated. Particularly, the pump injection rate is dependent on the injectivity of the well. Depending on the circumstances, the injection rate can vary during operation, as can the duration of operation. After Stage 5, the pumping rate must be increased and adjusted according to the maximum allowable pumping pressure (1841.72 psi) until the end of pumping.

**Figure 10:**
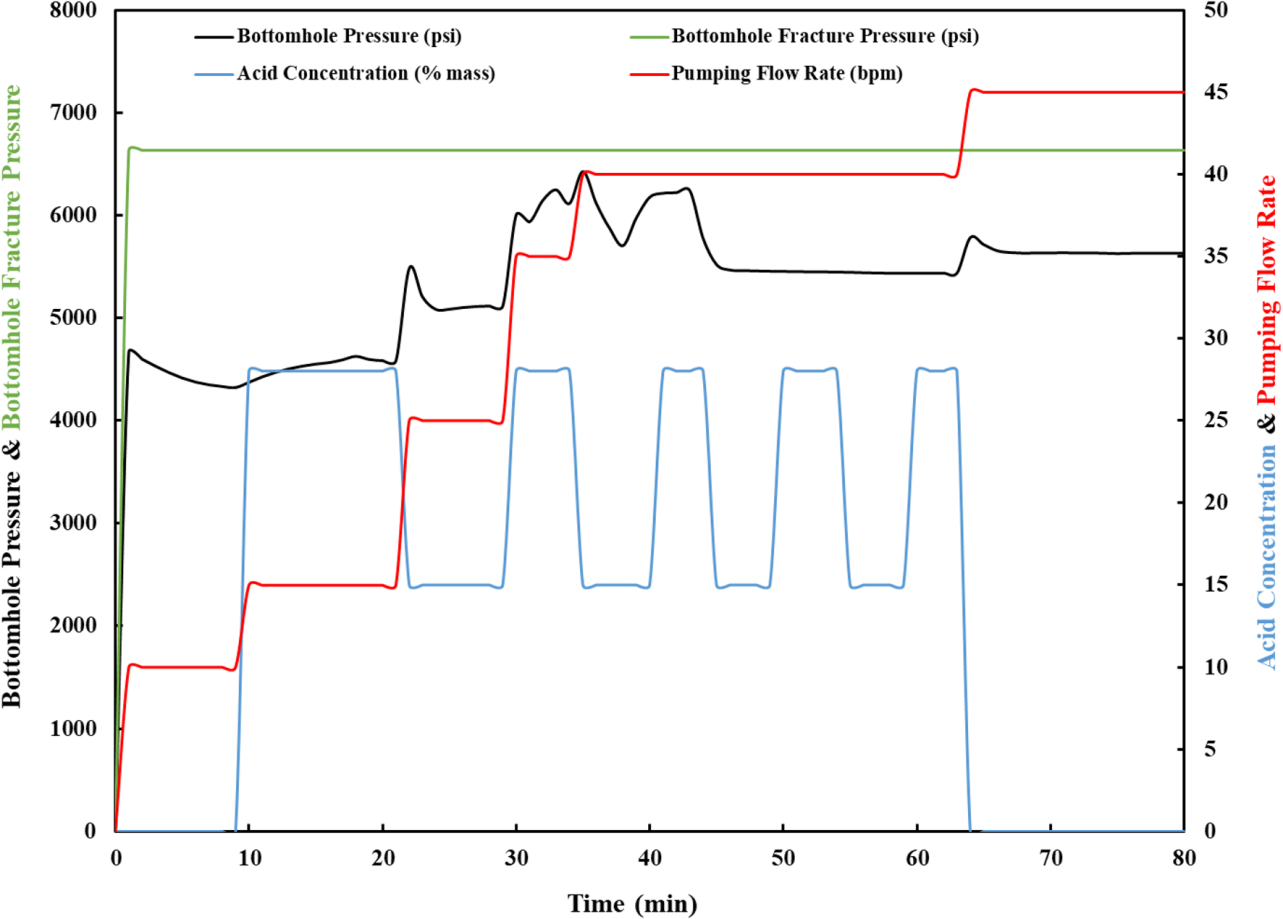
Pressure match during the acid injection.

Figure 10 depicts the simulation results for the acidizing pumping rate and pressure. Pumping activity is maintained below the fracture pressure throughout the twelve-stage pumping operation to prevent reservoir fracture. The observed changes in the pressure curve are the result of the VDA entering the reservoir and altering the fluid path into layers with lower permeability that require higher pressure to pump the solution.

**Figure 11:**
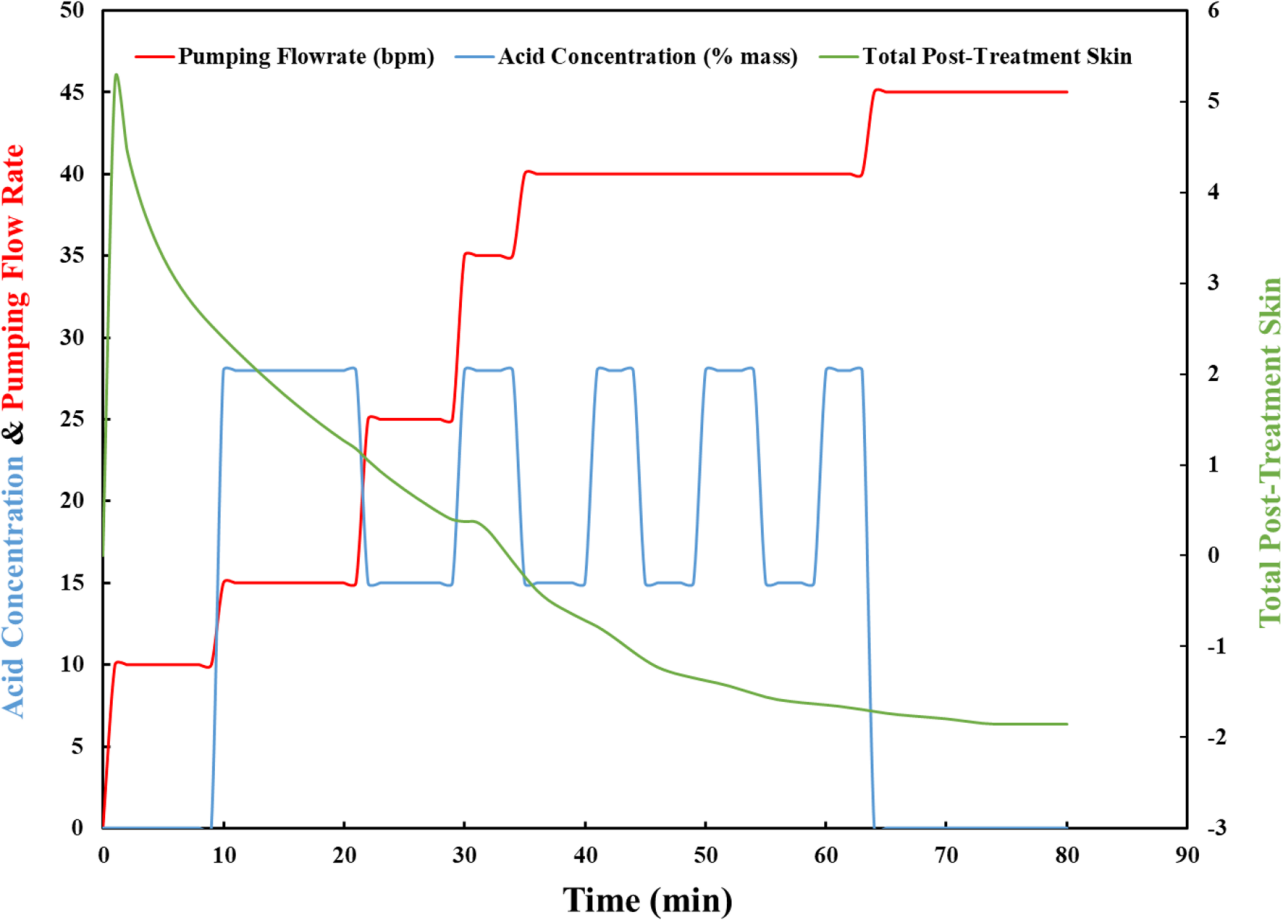
Skin reduction, pumping flow rate and acid concentration plot.

As shown in Fig. 11, when the main flush containing 28% HCl comes into contact with the formation, the skin starts to recede. This is due to the reaction of HCl with the formation's rock minerals. The same pattern is observed when the main acid flush is introduced in subsequent stages to produce additional skin reduction. This is due to the fact that when viscoelastic diverting acid (VDA) is introduced, the diverter enters the higher permeability layers and temporarily blocks them so that the other acid stages can treat the lower permeability zones. The final skin score for this case is −1.89, indicating that the treatment has a high potential for reducing skin damage.

**Figure 12:**
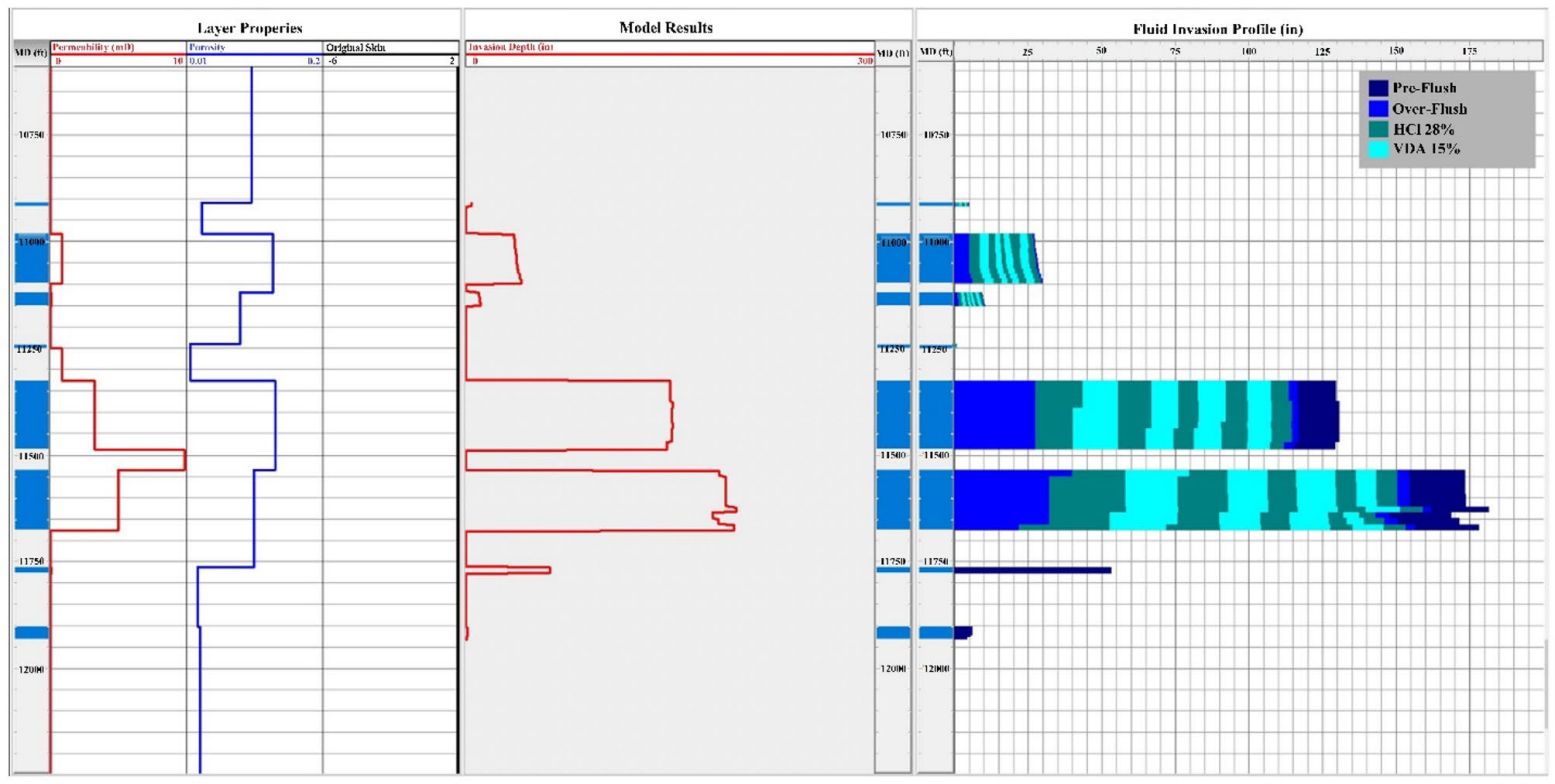
Acid invasion profile.

In addition, the acid invasion profile depicted in Fig. 12 reveals that the majority of the VDA flows into the layers between 11,300 and 11,600 feet, which have a higher permeability, and after a short time begins to increase the fluid's viscosity. This incident made it possible for later treatment stages to reach layers with lower permeability. As a result, all reservoir layers are stimulated, and maximum radial penetration is achieved up to 175 inches from the wellbore.

The simulation revealed that the matrix acidizing treatment increased the well's productivity by creating wormholes around the damaged area and increasing the formation permeability. The treatment also decreased the skin factor and improved the well flow efficiency. The acidizing of the matrix was designed and carried out in accordance with the best practices and recommendations for carbonate reservoirs.

### Operation

#### Oprteration procedure

**Figure 13:**
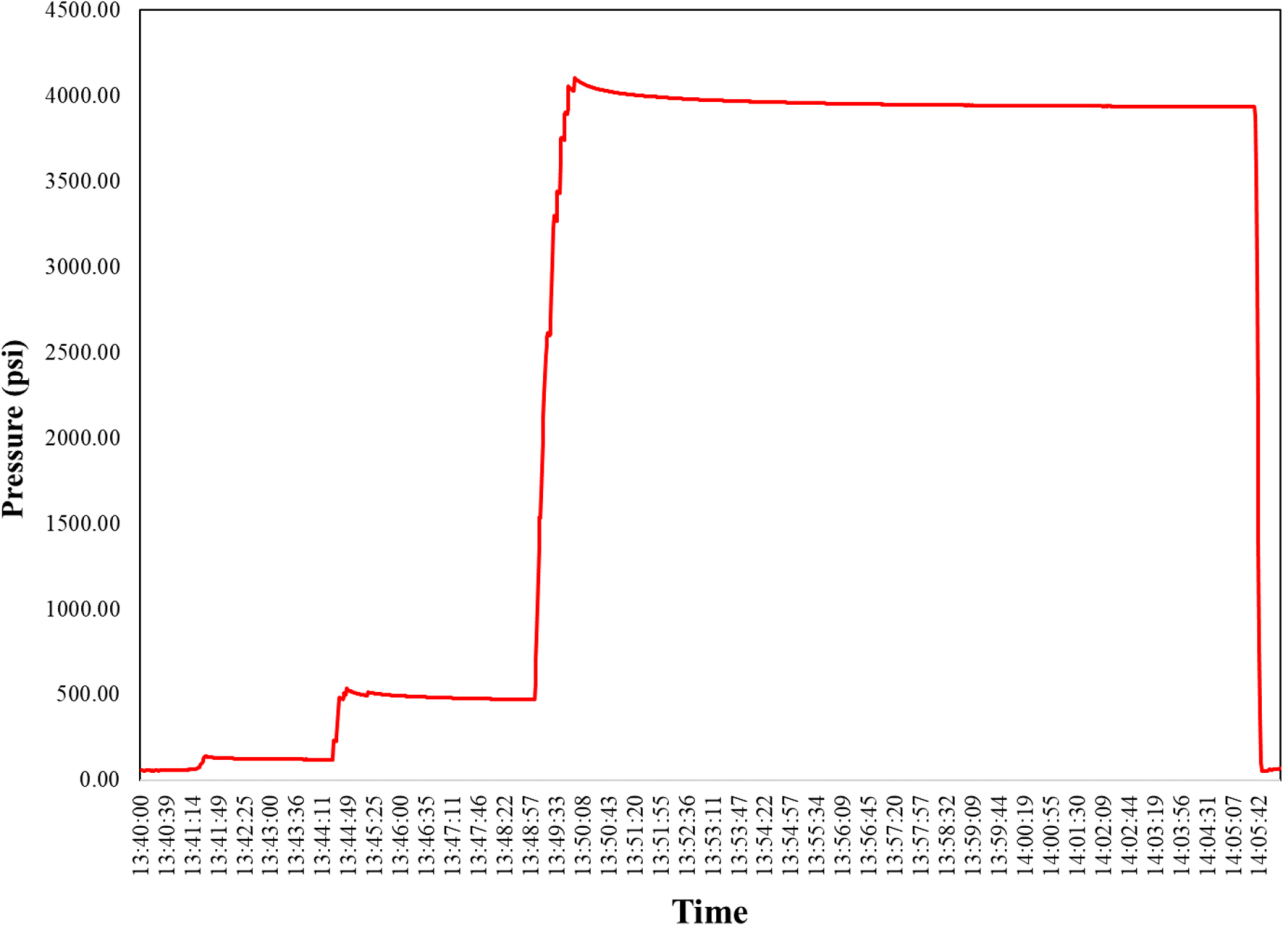
Pressure test before starting acid injection.

One of the main objectives of this project was to perform a successful acidizing operation on the desired reservoir using a five-step methodology. The first step of this methodology was to conduct a pressure test on the pipes, connections, and equipment before injecting acid into the well. This step was crucial to preventing any problems, such as acid leakage during the operation, that could compromise the safety and efficiency of the process. The pressure test was performed by applying a pressure higher than the operating pressure of acid injection for a certain period of time and monitoring the pressure drop along the route from the ship to the wellhead. The pressure test curve for this operation is shown in Fig. 13.

Figure 13 shows the results of the initial pressure test that was performed prior to the acidizing treatment. It can be seen that the pressure was gradually increased from zero to 500 psi and then held for five minutes. This was done to check the initial integrity of the equipment and detect any possible defects or flaws. The results show that there was no significant pressure drop during this stage, indicating that the equipment was in good condition and ready for further testing. The next stage of the test involved increasing the pressure from 500 to 4000 psi, which was the maximum pressure allowed for the operation. This was done to simulate the actual acidizing treatment and to verify that the equipment can handle the high-pressure environment without any problems. The results show that the pressure was stable at 4000 psi and that there was only a minimal pressure drop that was ignorable. This demonstrates that the equipment had high resistance and durability against the high-pressure acidizing treatment, and there was no risk of leakage or failure. Therefore, based on these results, it can be concluded that the equipment used for this project was suitable and reliable for performing the acidizing treatment at the desired pressure. The equipment had high pressure stability and did not show any signs of weakness or damage during the test. Moreover, the equipment did not cause any interference or disturbance to the reservoir conditions or properties due to its high compatibility and adaptability. Thus, the acidizing treatment was ready to be executed with confidence and safety.

#### Operation results

**Figure 14:**
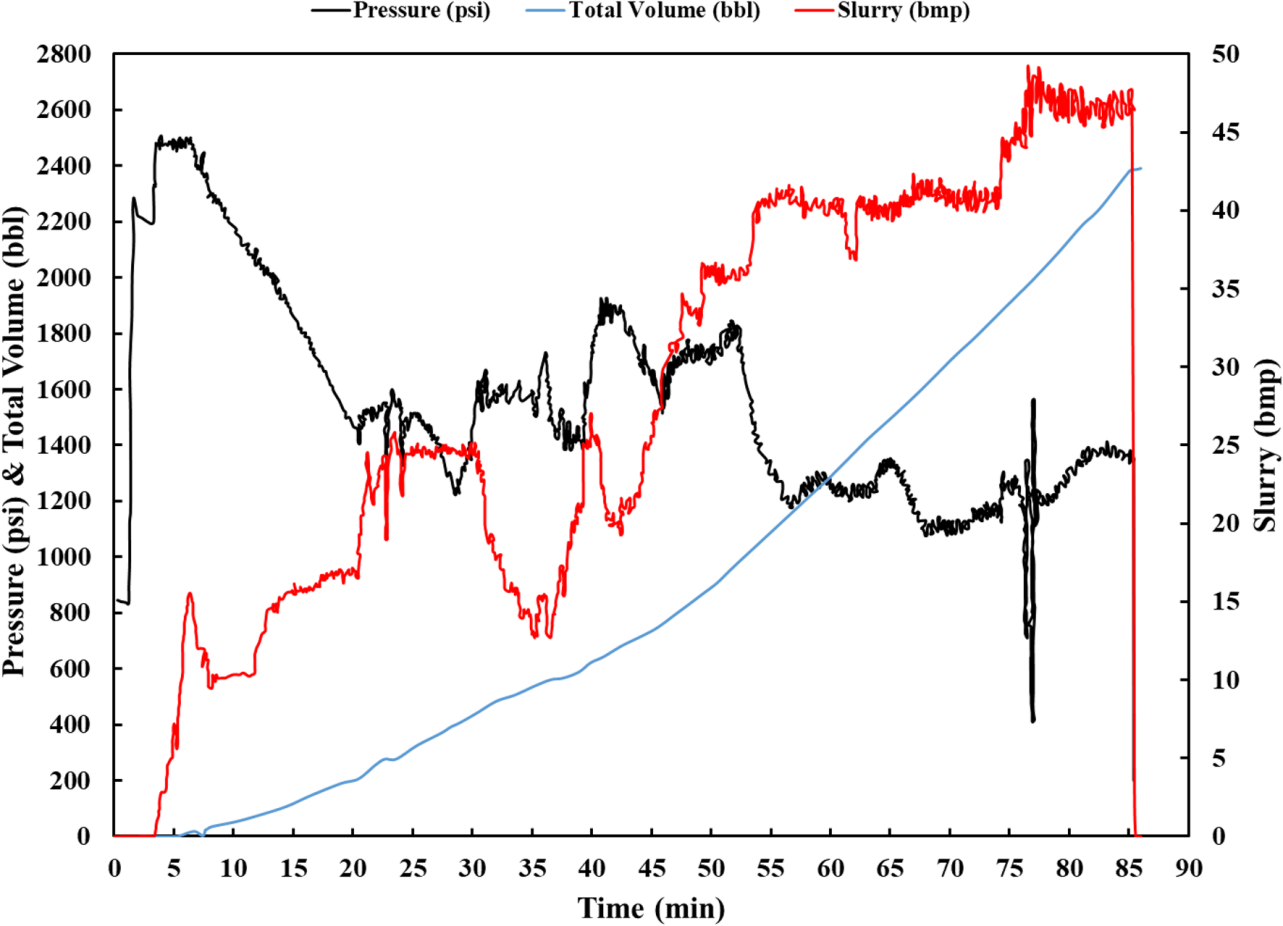
Pressure, volume and pump rate changes during the acid injection.

Figure 14 illustrates the pressure and volume of the injected acid during the operation.

As shown in Fig. 14, the surface pressure of the well was approximately 2500 psi at the start of the acid injection operation. The acid injection is performed by the bull-heading method in this project, and therefore, it takes about 22 min for the injected pre-flush to reach the perforated zone, according to the volume of the string and the fluid injection rate. This explains why the injection pressure curve, after a continuous decrease from the value of 2500 pam at the beginning of the injection, changes its trend to a value of around 1500 psi in the 22nd minute when it reaches the depth of the perforated zone and does not decrease any further. This phenomenon indicates that the injection fluid reaches the reservoir inlet. If the injection fluid enters the paths with high permeability, the injection pressure is reduced, and the operation can be continued with the pre-designed injection rate according to the simulation. The pressure variations that can be observed in the curve are due to the VDA reaching the reservoir. After that the VDA reacts with the reservoir rock and turns into a gel, the paths with high permeability are blocked, and the acid enters the paths with lower permeability, which increases the injection pressure.

As calculated in the previous section, there is a limit to the injection pressure when injecting acid into the reservoir. For instance, in some stages, such as the fourth to sixth stage, the injection flow rate is lower than the amount designed in the acidizing program. The reason for this is the reservoir’s resistance to acid injection, and therefore, to meet the maximum injection pressure, which is around 1840 psi, and prevent failure in the reservoir, the injection flow rate has to be reduced, which is done by reducing the engine speed of the injection pumps. It is important to note that during acid injection, available sensors evaluate pressure data, injection rate, and injected volume in real time and keep track of them throughout the injection. Therefore, if sudden changes in pressure are detected, they can be managed by controlling the injection rate. As mentioned in the previous section and according to the simulations that have been done, after reaching the sixth stage, the acid injection rate should reach its maximum allowed value according to the surface pressure limit. Since the surface pressure at this time is lower than the maximum injection pressure, from minute 48 onwards, acid injection is performed at the same rate as designed. After this period, the well is opened, and return flow is taken from it. For this purpose, the path from the wellhead to the burner boom has been opened so that fluids injected in the acidizing operation can be taken out of the well together with the produced gas.

Depending on reservoir pressure and production rate, it will take some time for all these fluids to leave the reservoir and well. In this project, after keeping the well open for 153 h and getting return flow from it, the main parameters of the well and reservoir such as production rate, pressure, temperature of the wellhead and BS&W have been measured, which can be seen in Table 15.

**Table 15:** Production parameters of the well.

Pre-acid clean up	Post-acid clean up
Choke Size: 72/64’’ fix	Choke Size: 72/64’’ fix
WHP: 1075 psia	WHP: 1982 psia
WHT: 132.8 ℉	WHT: 149 ℉
BS&W: 7%	BS&W: 3%
EsT. Rate: 26 MMSCF/D	EsT. Rate: 50 MMSCF/D NeT Flowing Time: 153 h

As shown in Table 15, the production rate of gas has increased significantly by 100%, which indicates a very high efficiency of the acidizing operation. Also, the pressure and temperature of the wellhead have increased after acid treatment, which are good signs of production enhancement after matrix stimulation. By increasing the WHP, it can be proven that the skin damage has decreased. In the previous section, it was shown that by stimulating the reservoir, the skin damage of the near wellbore will be decreased to about −1.89. While skin damage is decreased, the pressure drop near the wellbore will be increased, and as a result, the wellhead pressure will be enhanced. The increment of the pressure in the desired well is 907 psi (about 84%), as can be seen in Table 15. BS&W stands for Basic Sediments and Water. BS&W is a standard oil and gas industry term to describe unusable elements in a well stream. One of the major purposes of matrix acidizing is to increase the wellhead pressure and decrease BS&W. After flowing the well for 153 h, the BS&W has decreased from 7 to 3%, which is an important sign of high-performance stimulation. So, through raising the production rate and WHP and also reducing the BS&W, the main goals of carbonate matrix acidizing have been achieved in this project.

## Conclusion

This article has presented a comprehensive case study on the application of HCl and VDA systems for enhancing a gas well in a carbonate and dolomite reservoir in southern Iran. It demonstrates how laboratory tests, simulation tools, and field operations can be used to optimize well productivity and gas recovery processes. The findings of this study are as follows:

- This study has developed and optimized two acid solutions: 28% HCl and 15% VDA, specifically designed for the well under study. It has evaluated the performance of various additives in the acid solutions, such as corrosion inhibitors, non-emulsifiers, iron-sequestering agents, surfactants, and H_2_S scavengers. Explanation of the criteria and methods used to select the optimal additives and their concentrations for the acid solutions has also obtained from this article.
- This article has applicated a numerical model to simulate the acid injection and fluid flow in the reservoir, and has demonstrated that the most optimal method of acidizing in this operation is the use of five main acid injection stages and five VDA injection stages. It can be obtained from the paper that the skin effect starts to decrease immediately after the acid injection and reaches from 5 to −1.89.
- This paper has reported the results and outcomes of the field operation involving the injection of the acid solution into the well. It also has shown that the use of the VDA system and the bull-heading method are effective in diverting the acid into the low permeability layers and stimulating the whole reservoir. The article also indicates that the proposed combination in this project has caused a 100% increase in gas production, an 84% increase in the pressure of the produced gas, as well as a decrease in the amount of BS&W from 7 to 3%.

The study concludes that the use of HCl and VDA systems can significantly improve well performance and gas recovery in carbonate reservoirs. It emphasizes the significance of laboratory tests, simulation tools, and field operations in the design and implementation of effective acidizing treatments. This research contributes to the existing body of knowledge on matrix acidizing techniques and provides valuable information for future applications in similar reservoirs.

## Recommendations

- Real-Time Monitoring: A real-time monitoring system should be developed to track the performance of VDA during operations. This system should provide immediate feedback on the effectiveness of the acidizing process, allowing for timely adjustments and optimization.
- Unbalanced Staging: Implement unbalanced staging in acidizing operations, using geological information and area logs. This approach can help target specific zones more effectively and adapt the acidizing treatment to the unique characteristics of each reservoir.
- Machine Learning Models: Several machine learning models could be developed in order to plan and execute the staging of acidizing operations. These models can analyze vast amounts of data to predict outcomes, optimize treatment plans, and enhance decision-making processes.

## Data Availability

All data generated or analysed during this study are included in this published article.

## List of symbols

VDA: Viscoelastic diverting acid
HCl: Hydrochloric acid
H_2_S: Hydrogen sulfide
HF: Hydrofluoric acid
FeCl_3_: Ferric chloride
pH: Potential of hydrogen
Fe(OH)_3_: Iron hydroxide
CR: Corrosion rate
CO_2_: Carbon dioxide
ppm: Part per million
bbl: Barrel
Bpm: Barrel per minute
BS&W: Basic sediments and water
WHP: Well head pressure
$$\rho$$: Density
W_loss_: Weight loss
FeS: Ferrous sulfide
FeCl_2_: Ferrous chloride
CdSO_4_: Cadmium sulfate
CdS: Cadmium sulfide
H_2_SO_4_: Sulfuric acid
ID: Inner Diameter
OD: Outer diameter
TVD: True vertical depth
MD: Measured depth
MSIP: Maximum surface injection pressure
WHT: Well head temparature
MMSCF/D: Million standard cubic feet per day
